# Patient, family and productivity costs of end-stage renal disease in the Netherlands; exposing non-healthcare related costs

**DOI:** 10.1186/s12882-021-02548-y

**Published:** 2021-10-16

**Authors:** Eline F. de Vries, Jeanine Los, G. Ardine de Wit, Leona Hakkaart - van Roijen

**Affiliations:** 1 Department of Quality of Care and Health Economics, Centre for Nutrition, Prevention and Health Services, National Institute for Public Health and the Environment, PO Box 1, 3720 BA Bilthoven, The Netherlands; 2 Erasmus School of Health Policy & Management (ESHPM), Institute for Medical Technology Assessment (iMTA), Erasmus University Rotterdam, Rotterdam, The Netherlands; 3 Julius Centre for Health Sciences and Primary Care, University Medical Centre Utrecht, Utrecht University, Utrecht, The Netherlands

**Keywords:** Patient- and family costs, Dialysis, Kidney transplantation, Informal care, Productivity loss

## Abstract

**Background:**

Healthcare costs related to ESRD are well-described, but broader societal costs of ESRD are less known. This study aimed to estimate patient and family costs, including informal care costs and out-of-pocket costs, and costs due to productivity loss related to ESRD, for patients receiving dialysis and living with a kidney transplant, using a bottom-up approach.

**Methods:**

A total of 655 patients were asked to complete a digital questionnaire consisting of two standardised instruments (iMCQ and iPCQ) from November 2016 through January 2017. We applied a retrospective bottom-up cost estimation by combining data from the questionnaire with unit prices from the Dutch costing manual.

**Results:**

Our study sample consisted of 230 patients, of which 165 were kidney transplant recipients and 65 received dialysis. The total annual non-healthcare related costs were estimated at €8284 (SD: €14,266) for transplant recipients and €23,488 (SD: €39,434) for dialysis patients. Costs due to productivity loss contributed most to the total non-healthcare costs (66% for transplant recipients and 65% for dialysis patients), followed by informal care costs (26% resp. 29%) and out-of-pocket costs, such as medication and travel expenses (8% resp. 6%).

**Conclusion:**

By exposing patient, family and productivity costs, our study revealed that dialysis and transplantation are not only costly within the healthcare system, but also incur high non-healthcare costs (18–23% resp. 35% of the total societal costs). It is important to reveal these types of non-healthcare costs in order to understand the full burden of ESRD for society and the potential impact of new therapies.

## Background

The disease burden of end-stage renal disease (ESRD) is one of the highest worldwide [1]. In the Netherlands, the number of prevalent ESRD patients continues to rise, despite stabilization of incidence rates. This is mainly due to an increased life-expectancy of ESRD patients through better access to kidney dialysis and kidney transplantation [2–4]. In the last decennium, the number of patients with ESRD depending on renal replacement therapy (RRT) has risen with approximately 28% (from 13,994 patients in 2009 to 17,933 in 2019) [2, 5]. As a consequence, the impact of ESRD on the national healthcare budget is increasing as well. Estimates show that approximately 1% of the Dutch healthcare budget was spent on RTT in 2017 [6].

The economic impact of ESRD stretches further than the healthcare budget alone. For example, ESRD has a major impact on the patients’ productivity, both related to an impaired health status and the time-consuming and intensive character of dialysis. Approximately 70% of the ESRD patients are unemployed [7] and even when ESRD patients are capable to maintain their work, society faces substantial costs due to absenteeism and presentism (i.e., being less productive while at work) [4]. Furthermore, ESRD patients are often highly dependent on relatives in their daily activities. Besides the potential risk of health issues for informal caregivers, time consumed by caregiving cannot be used for other activities such as paid work or leisure and therefore can be a substantial cost to society [8, 9].

Because of the considerable impact ESRD has on society, it is important to incorporate *all* relevant costs, thus not limited to the healthcare system, in economic evaluations [10–12]. This is referred to as the ‘societal perspective’ [13]. In general, societal costs can be distinguished in healthcare related costs, patient- and family costs and other costs, the latter including costs of productivity losses. Only by adopting a societal perspective, the question underlying economic evaluations, i.e. whether social welfare will improve from the introduction of a specific health service or intervention, can be answered [14, 15].

Despite the fact that an increasing number of economic evaluations claims to have taken a societal perspective (e.g. [11, 16]), many studies lack patient-level observational data on the non-healthcare costs [4, 17], as these typically consist of costs that are not easily retrievable from national data bases or hospital administration. Only by revealing non-healthcare related costs as observed in detail at the patient level (i.e. bottom-up approach), the full burden of ESRD for society, as well as the potential economic effects of new therapies can be understood. Therefore, this study aimed to estimate the non-healthcare costs related to ESRD in a bottom-up approach, as a supplement to the recent study of Mohnen, Los [18], who estimated healthcare related costs of ESRD using Dutch claims data in a top-down approach. Specifically, we aimed to estimate patient and family costs, including informal care costs, out-of-pocket costs, and costs due to productivity loss for ESRD patients receiving dialysis and living with a kidney transplant.

## Methods

### Study population

**Fig. 1:**
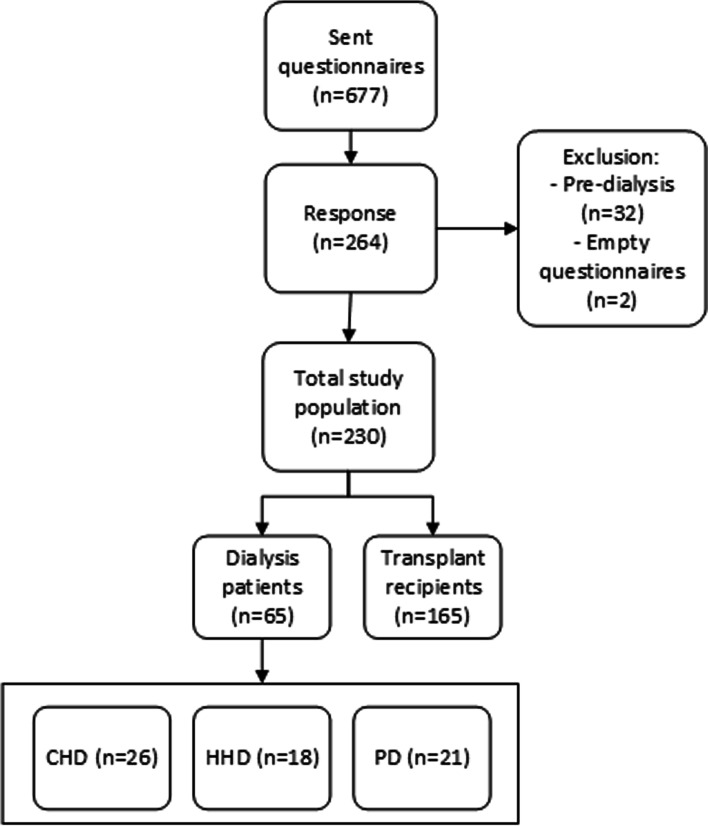
Flowchart study population. CHD: haemodialysis in-centre; HHD: haemodialysis at home; PD: peritoneal dialysis

We sent 677 RRT patients digital questionnaires for the purpose of our study (Fig. 1). We approached the patients through the Dutch Kidney Patients Association and a dialysis centre with a large population of home-based therapies (VieCuri dialysis centre in Venlo, the Netherlands). The patients from the Kidney Patients Association of the Netherlands participate in a panel that are frequently consulted for research purposes. The response to our questionnaire was 39% (*n* = 264), whereof 5% was excluded from further analysis, either because the respondent was still pre-dialysis (*n* = 32) or because of empty cases (*n* = 2).

The distribution of dialysis patients and transplant recipients in the approached group was highly skewed, with relatively more transplant recipients. Although this distribution of treatment modalities complies with the real-world situation in the Netherlands, the total number of dialysis patients was too small to enable subgroup analysis for all dialysis modalities (peritoneal dialysis (PD), haemodialysis in-centre (CHD) or haemodialysis at home (HHD)). Therefore, we divided our study population in the following main RRT modalities: 1) patients who receive dialysis, and 2) kidney transplant recipients.

### Data collection

We sent a digital questionnaire consisting of two standardised instruments to the patients. The iMTA Medical Consumption Questionnaire (iMCQ) is an instrument to collect formal and informal care service use and out-of-pocket expenses [19]. For the current study, questions of the iMCQ about informal care, travel expenses and out-of-pocket costs were selected and included in the questionnaire. In addition, respondents completed the iMTA Productivity Cost Questionnaire (iPCQ). The iPCQ is a validated tool to measure absence from work, presenteeism and productivity losses from unpaid work, e.g. household or volunteer work [20]. Absence from work is measured by the number of days lost from paid work due to illness. Presenteeism refers to productivity loss due to illness while being at work (sub-optimal productivity). The module of the iPCQ to measure presenteeism consists of three questions. Firstly, patients were asked whether they suffered from health problems at work and if so, for how many days. Finally, patients were asked to rate their work performance on these days compared to their functioning on normal working days using a 10-point rating scale. Questions about informal care and out-of-pocket costs were about the last 3 months and work-related questions about the previous 4 weeks, which is in accordance with the default recall period of the iPCQ.. Data on long-term absence from work (extending the last 4 weeks) was collected in a separate question. Following the Dutch costing manual, we extrapolated the observed three-month data on informal care, out of pocket payments and travel costs and one-month absence from work data to 1 year [19–21]. The study was carried out in accordance with all relevant (Dutch) guidelines and regulations. The iMCQ and the iPCQ can be requested through https://www.imta.nl/questionnaires/.

### Cost components

We applied a bottom-up cost estimation by multiplying the data on the number of hours of informal care, travel expenses and productivity costs at the individual patient level with unit prices from the Dutch costing manual [21]. Table 1 shows the relevant cost components and associated unit prices.

**Table 1:** Cost components and associated unit costs in 2016 euro’s

Cost component	Cost per unit €
**Patient & family related costs**	
Informal care (hour)	14
Co-payments medication/formal home care	Based on real expenses
*Travel expenses*	
Car or public transport (per km)^a^	0.19
Taxi (per km) + €3 start tariff	2.68
Parking (per day)	Based on real expenses
**Productivity costs**	
Paid work men (hour) ^b^	38
Paid work women (hour) ^b^	32
Unpaid work (hour)	14

^a^Including gasoline, maintenance and depreciation costs of car; ^b^Friction period is 85 calendar days (12 weeks)

#### Patient and family-related costs

Although patient and family costs are directly associated with the disease or treatment, they occur outside the formal healthcare system. The main driver of these costs is usually informal caregiving, whereby relatives take over certain tasks of the patient who is no longer able to perform the tasks himself due to the disease. In this study, informal care concerns household or practical support (e.g. financial matters), assistance in personal care or treatment (e.g. medication), or transport. According to the Dutch costing manual the costs of informal care were calculated based on the substitution value method, which values time spent by informal caregivers according to professionals’ wage rates for household work [21].

Beside the costs of informal care, we also took into account the out-of-pocket expenses by the patient or family. First, we included patients’ spending on medication or formal home care that was not covered by health insurance. Second, travel expenses associated with medical treatment or appointments with the General Practitioner (GP) or medical specialist was measured. Depending on the type of transport, the usage costs were calculated based on the average distance from home to the GP or hospital combined with the average fuel cost per kilometre or taxi costs (according to the Dutch costing manual) and the number of visits per year. The parking costs were considered for appointments in the hospital only. Usually a special regulation (no parking costs) applies to patients undergoing dialysis treatment in the hospital.

#### Productivity costs

Productivity losses comprise the costs associated with absenteeism, presenteeism and reduced participation in unpaid work activities. Absenteeism for paid work was measured using the friction-cost method [21]. The friction costs method allows for a more realistic estimate of the productivity costs and is the recommended methodology of the Dutch Costing manual for health economic studies [21]. Applying the friction cost method assumes that productivity costs are limited to the time to replace an absent worker. The friction period is 12 weeks, in accordance with the Dutch Costing manual [21]. To calculate the costs of productivity losses, hourly wage costs (Table 1), adjusted for gender, were obtained from the Dutch costing manual [21] and converted to reflect 2016 prices. Wage costs refers to gross salaries, augmented with social insurance premiums paid by employers. The costs for presenteeism (i.e. decreased productivity while at work) were calculated by extrapolating the hours of being not productive in the last 4 weeks to hours on a yearly basis multiplied by the hourly wage costs (Table 1).

Unpaid work includes voluntary work and also household activities, such as cooking, cleaning and gardening and is valuated at professionals’ wage rates for housekeeping [21].

### Statistical analyses

We calculated the mean and standard deviation (SD) of the units or the costs per variable over all respondents. In addition, frequency distributions were used to present the number of patients per modality and the number of patients who used a certain type of care (e.g. number of patients who used informal home care). For the limited number of (non-structural) missing values (0–3,5%), the mean of the observed user values per modality was imputed. The Mann-Whitney test was used to assess differences between modality for continuous variables and the Pearson Chi-Square test for the categorical variables. All prices were reported in Euros and converted to the year 2016 according to the Dutch Consumer Price Index (2014 to 2016: 1.009).

## Results

### Patient characteristics

Table 2 reports the patient characteristics of the study sample compared to all RRT patients in the Netherlands (Dutch Renal Registry [5]). The final study sample included 230 patients, of whom 165 were transplant patients (of whom 80% received the graft more than 2 years ago) and 65 received dialysis. The dialysis group consisted of 26 CHD patients, 18 HHD patients and 21 PD patients. The study sample consisted of relatively more patients living with a transplant than in the Dutch population (71.7% in the study sample versus 63% in the Dutch population) and dialysis patients in the study sample tended to be younger than in the Dutch population (on average 56.5 in the study sample and 67 in the Dutch population) [5].

**Table 2:** Patient characteristics

	***Study sample***		***Dutch population***^a^	
	*Transplant recipients*	*Dialysis patients*	*Transplant recipients*	*Dialysis patients*
*Patient characteristics*				
N (%)	165 (72)	65 (28)	10,812 (63)	6320 (37)
Age, mean (SD)	58 (13)	57 (13)	56 (15)	67 (15)
Gender (% men)	50	57	–	–
Marital status (% with partner)	80	74	–	–
% employed	30	20	–	–

^a^based on figures from the Dutch Renal Registry [5]

### Costs of informal care

**Fig. 2:**
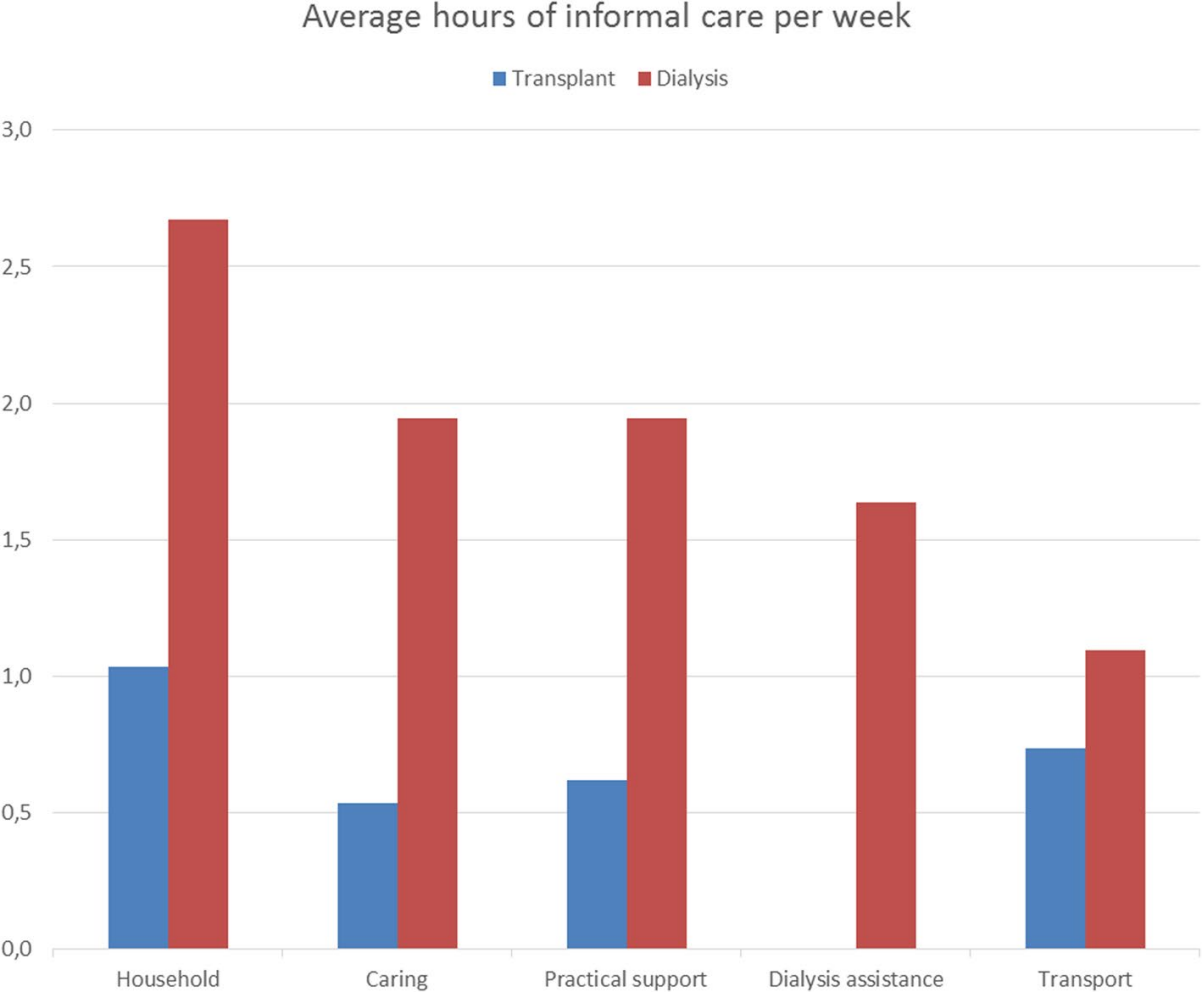
Hours of several types of informal care per week (averaged over three months)

Figure 2 shows the weekly hours of informal care received by ESRD patients, as averaged over all patients. Overall, the hours of all types of informal care per week was significantly higher among the patients who received dialysis (*P* < 0.001) compared to transplant recipients. The difference is in part related to the timing of the questionnaire versus transplantation: 90% of the transplant recipient respondents received their graft more than 1 year prior to filling out the questionnaire. Because our sample consisted most stable transplant recipients, we found a lower percentage of patients that used informal care among the transplant recipients (Table 3). Moreover, the category ‘dialysis assistance’ was only applicable to dialysis patients and therefore no comparison could be made with transplant recipients. A similarity between the two modalities was that most hours of informal care was spent on household activities (on average 1.0 h per week for transplant recipients vs 2.7 h for dialysis patients).

**Table 3:** Mean annual informal care costs per modality in 2016 euro’s

	***Transplant recipients (n = 165)***			***Dialysis patients (n = 65)***		
	*Mean costs (SD)*		*% users*	*Mean costs (SD)*		*% users*
*Informal care*						
Household^a^	759	(2385)	25	1964	(3724)	37
Caring^a^	393	(1731)	8	1430	(3974)	23
Practical support^b^	454	(1802)	13	1430	(3794)	32
Dialysis assistance	0	(0)	0	1197	(3379)	23
Transport^a^	541	(2035)	27	811	(1883)	43
**Total costs informal care**^b^	**2147**	**(6571)**	**38**	**6832**	**(12631)**	**68**

^a^significant difference for alpha < 0.05; ^b^ significant difference for alpha < 0.01

Table 3 shows the mean annual costs of informal care per modality. The percentage of patients that used certain types of informal care was lower compared to the sum of the partial percentages, because some patients had received multiple types of informal care. The dialysis patients had significant higher costs for informal home care (€6832) compared to the transplant recipients (€2147). Even when the costs for dialysis assistance were filtered out, the informal care costs remained 2.5 times higher for dialysis.

### Out-of-pocket costs

Table 4 shows the mean annual out-of-pocket costs of patients and family per modality. These costs were distinguished in co-payments for formal home care and medication, and transportation costs. The out-of-pocket cost were predominantly defined by co-payments for medication, which was more often applicable and significantly higher (€989 vs €422) for patients receiving dialysis, compared to transplant recipients. In addition, dialysis patients spent a significantly higher amount on both gasoline and taxi’s, and parking (€176 vs 398). Only the co-payment for formal home care was slightly higher (€75 vs €61) for transplant recipients. Overall, the mean annual out-pocket costs were significantly higher for dialysis patients than for transplant recipients.

**Table 4:** Mean annual out-of-pocket costs of patient and family per modality in 2016 euros

	***Transplant recipients (n = 165)***			***Dialysis patients (n = 65)***		
	*Mean costs (SD)*		*% users*	*Mean costs (SD)*		*% users*
*Co-payments*						
Formal home care	75	(417)	5	61	(246)	8
Medication^a^	422	(1086)	34	989	(1488)	65
**Total co-payments**^a^	**497**	**(1154)**		**1050**	**(1504)**	
*Transportation*						
Gasoline/taxi^a^	137	(495)	88	298	(4980)	91
Parking^a^	39	(82)	64	100	(368)	40
**Total transportation**^a^	**176**	**(499)**		**398**	**(607)**	
**Total out-of-pocket costs**^a^	**673**	**(1294)**		**1448**	**(1628)**	

^a^significant difference for alpha < 0.05

### Productivity costs

The employment rate among transplant recipients and dialysis patients was 30 and 20%, respectively (Table 2). Absence from work, of which most was short-term, was reported by 24% of the working transplant recipients (7% of 30%) and 62% of the working dialysis patients (12% of 20%) (Table 5). Extrapolated to an annual base, an average of five absence days for transplantation recipients were reported, which corresponds with annual productivity losses of €1185. For dialysis patients, the average annual number of days absent from work was slightly higher (6 days), leading to about €1300 of productivity loss per year.

**Table 5:** Mean annual productivity costs in 2016 euro’s

	***Transplant recipients (n = 165)***			***Dialysis patients (n = 65)***		
Productivity costs	*Mean (SD)*		*% users*	*Mean (SD)*		*% users*
*Absenteeism*^a, b^						
Days less worked	5	(20)	7	6	(18)	12
Costs absenteeism in €	1185	(5372)		1302	(4314)	
*Presenteeism*^a^						
Hours less worked	33	(117)	12	85	(409)	12
Costs presenteeism in €	1160	(4080)		3231	(15631)	
*Unpaid/voluntary work*						
Average hours less worked	223	(577)	41	763	(1351)	52
Costs of missed unpaid work in €^a^	3120	(8080)		10,675	(18918)	
**Total**	**5464**	**(10754)**		**15,208**	**(29706)**	

^a^Employment rate for transplant recipients was 30% and for dialysis patients was 20% ^b^calculated using the friction method (costs include max 12 weeks of paid work)

Presenteeism caused on average 33 h production loss per patient per year for transplantation recipients, resulting in €1160 per patient per year. The costs of presenteeism for the dialysis group was €3231, which is substantially higher compared to the transplant group.

The costs of reduced productivity in unpaid/voluntary work was significantly higher for the dialysis patients (€10,675) compared to the transplant recipients (€3120). Within the dialysis group, 52% of the patients reported having problems with unpaid work as household activities or voluntary work. This percentage was 41% in the group of transplant recipients. Averaged over all patients, the number of missed hours of unpaid work was about 3.5 times higher in dialysis patients than in transplant recipients.

Table 6 gives an overview of the total patient and family related cost (i.e. informal care and out-of-pocket expenses) as well as the costs due to productivity losses. Overall, the non-healthcare costs of the dialysis group were significant higher compared to the patients with a transplant.

**Table 6:** Total mean annual patient, family and productivity costs in 2016 euro’s

	***Transplant recipients (n = 165)***		***Dialysis patients (n = 165)***	
	*Mean costs*	*(SD)*	*Mean costs*	*(SD)*
Total informal care costs	2147	(6571)	6832	(12631)
Total out-of-pocket costs	673	(1294)	1448	(1628)
Total productivity costs	5464	(10754)	15,208	(29706)
**Total non-healthcare costs**	**8284**	**(14266)**	**23,488**	**(39434)**

## Discussion

This is one of first studies that investigated the non-healthcare costs of ESRD by estimating informal care costs, out-of-pocket costs as well as costs due to productivity losses using a bottom-up approach in 2016. We estimated the mean total annual non-healthcare related costs at €8284 for transplant recipients and €23,488 for dialysis patients. Costs due to productivity loss contributed most to the total non-healthcare costs 66% for transplant recipients and 65% for dialysis patients), followed by costs related to informal care and out-of-pocket costs, such as medication and travel expenses. In all cost categories, costs among dialysis patients exceeded those of kidney transplant recipients.

Our study was performed supplementary to the top-down cost estimation of healthcare costs from Mohnen, Los [18], which was based on nationwide health claims data. By additionally estimating costs of productivity losses, out-of-pocket expenses and informal caregiving in the present study, we built a comprehensive overview of societal ESRD costs for the Netherlands. Combining figures from Mohnen, Los [18] and the present study, we estimate the total annual mean societal costs related to ESRD ranging from €101,752 to €130,273 depending on the treatment modality for dialysis patients and €23,437 for transplant recipients as from the second year after transplantation in the Netherlands in 2016. Within this estimate of total societal costs, non-healthcare costs contribute for approximately 18–23% for dialysis patients and 35% for transplantation recipients to the total costs related to ESRD.

Other studies estimating non-healthcare related ESRD costs using a bottom-up approach are few [10, 22–24] and none of them covered costs of productivity losses, out-of-pocket expenses and costs of informal care altogether for both dialysis patients and transplant recipients. When comparing estimations of the previous studies with our study, we see similar cost estimations for out-of-pocket expenses and informal care giving in dialysis patients from Hong Kong [22]. However, our study showed higher costs of informal care giving as compared to dialysis patients and kidney recipients and higher productivity costs in transplant recipients in a study comparing Danish, Norwegian, Swedish and Finnish data [24]. These differences may be due to variations in included cost categories across countries. For example, following the Dutch guideline for economic evaluations [20, 21] we included unpaid work (voluntary work and household activities, such as cooking, cleaning and nursing) in the estimation of productivity losses, while Eriksson, Karlsson [24] did not. Our results showed that productivity loss due to unpaid work covered about two third of the total productivity costs in dialysis patients which could largely explain the difference between Eriksson, Karlsson [24] and the present study.

The production losses were valued using the average wage by gender, in accordance with the Dutch costing manual. This approach is the most common applied method to increase the generalizability of the study. Given the relatively old age of our study population, this may be an underestimate, as the average Dutch worker is younger than 58. Another reason that we might have underestimated productivity costs, is that we valued production losses by applying the friction cost method [21]. This method prescribes to include only short term absenteeism based on the assumption that someone is replaced at work after a period of 12 weeks [25]. However, alternative valuation following the human capital method, as used in other countries, would have increased our estimates of productivity losses dramatically, as this method monetarizes the potential productivity loss of a person e (e.g. up to the age of 65–67) and both treatment modalities have high numbers of patients without formal employment, compared to the general population.

Our study has several limitations. First, the number of respondents in the survey was relatively small and highly skewed with relatively more transplant recipients. Comparing our study sample with Dutch Renal Registry data showed that the distribution over treatment modalities roughly complies with the real-world situation in the Netherlands. However, patients receiving dialysis in our sample were substantially younger compared to patients in the Dutch Renal Registry data [5]. This could have underestimated our calculations for the non-healthcare cost of dialysis, because older persons with ESRD generally experience more problems in daily live activities and may need more help from family members.

Second, due to the small number of dialysis respondents, it was not possible to conduct subgroup analysis for different dialysis modalities (peritoneal dialysis, haemodialysis in-centre or haemodialysis at home). It is likely that depending on dialysis modality, patients show differing patterns of costs outside the healthcare system.

Third, we did not include the health status of patients in our study. This is important as it influences the non-healthcare costs through the need for informal care and through an increased likelihood of productivity losses with impaired health state. Further research should be employed to address different dialysis modalities and health status in bottom-up cost estimation of non-health care related ESRD costs.

## Conclusions

By exposing costs outside the healthcare system, our study reveals that dialysis and transplantation are not only costly within the healthcare system, but also incur high non-healthcare costs suffered by patients and family. It is important to reveal these types of non-healthcare costs in order to understand the full burden of ESRD for society and the potential impact of new therapies.

## Data Availability

The data underlying this article will be shared on reasonable request to the corresponding author.

## Abbreviations

CHD: Haemodialysis in-centre
ESRD: End-stage renal disease
GP: General Practitioner
HHD: Haemodialysis at home
iMCQ: iMTA Medical Consumption Questionnaire
iPCQ: iMTA Productivity Cost Questionnaire
PD: Peritoneal dialysis
RTT: Renal replacement therapy
SD: Standard deviation
